# A qualitative study exploring the primary health care nurses’ lived experiences of cervical cancer screening

**DOI:** 10.4102/phcfm.v18i1.4986

**Published:** 2026-01-26

**Authors:** Maliketso G. Polane, Siyabonga B. Dlamini

**Affiliations:** 1 Department of Public Health Medicine, College of Health Sciences, University of KwaZulu-Natal, Durban, South Africa; 2 Cancer and Infectious Diseases Epidemiology Research Unit, College of Health Sciences, University of KwaZulu-Natal, Durban, South Africa

**Keywords:** cervical cancer screening, nurses’ screening experiences, negative experiences, positive experiences, subsequent screening

## Abstract

**Background:**

In the low- and middle-income countries (LMICs), cervical cancer is the primary cause of cancer-related female deaths. Lesotho has a cervical cancer incidence that is 2.7 times higher than the global average. This disease can be prevented by vaccination, which is not available in all countries, and through screening, which has been shown to have low uptake even among health professionals.

**Aim:**

To explore primary health care (PHC) nurses’ lived experiences of cervical cancer screening in Lesotho. Thus, to better understand how these experiences can impact nurses’ subsequent screening or their goal of offering screening services to their patients.

**Setting:**

The study was conducted at four PHC centres in the selected district.

**Methods:**

This is a qualitative study in which a phenomenological design was used. Ten nurses were purposively selected and participated in the in-depth interviews. The data were analysed thematically.

**Results:**

The findings revealed participants’ positive and negative experiences with cervical cancer screening. Positive experiences, including good nursing services, freedom of choice and pain prevention, influenced the nurses’ intentions to continue to test in the future and to strongly recommend screening to other women. The negative experiences included a lack of privacy, the absence of counselling and receiving results late. These negative experiences had a detrimental effect on nurses’ intentions to undergo screening in the future. However, they had no impact on their goal of offering screening to other women.

**Conclusion:**

The findings provided essential insights into why cervical cancer screening uptake is low among nurses.

**Contribution:**

This evidence is critical for augmenting the knowledge of how cervical cancer screening programmes could be improved to enhance nurses’ screening uptake and invariably facilitate higher screening rates within the general population.

## Background

Cervical cancer occurs when cells in the transformation zone of the cervix grow abnormally.^1^ Cancer of the cervix is a global health problem, ranking number two of all cancers that affect women of reproductive age, and the second most common fatal cancer in women,^2^ accounting for an incidence estimated at 527 624 women yearly and 265 672 deaths worldwide.^3^ Moreover, Hull et al.^4^ report that most of these cases, ranging between 84% and 90%, occur in low- and middle-income countries (LMICs).

Globally, breast cancer is the leading cause of cancer-related deaths among women.^5^ However, in Lesotho, cervical cancer incidence and mortality rank at the top.^6^ Moreover, there is a high cervical cancer burden in LMICs, with an age-standardised incidence rate of 75/100 000 women in the highest-risk countries and less than 10/100 000 women in the lowest-risk countries.^7^ Yet, the average coverage of cervical cancer screening in the majority of LMICs is only 19%, compared with 63% in high-income countries (HICs), demonstrating extremely low cervical cancer screening coverage in LMICs.^8^

Screening is the gold standard for controlling the disease.^8^ Yet, the general population, including healthcare workers (HCWs), has been observed to request cervical cancer screening via pap smear or any other screening method relatively infrequently. For instance, a study conducted in Nigeria on perceptions, attitudes and practices of cervical cancer screening among female doctors and nurses found that 98.1% were aware of it, 87.2% had satisfactory knowledge regarding the screening, but 12.8% had never been screened.^9^

In 2022, among women of all ages in Lesotho (*n* = 1 100 612), there were 598 new cases of cervical cancer and 413 deaths.^10^ Suantika et al.^11^ reported a study that involved nurses, which indicated that cervical cancer incidence was also a challenge among health professionals, particularly nurses. As nurses play a significant role in educating the public about the availability and necessity of cervical cancer screening services, they are constantly viewed as role models on health-related issues.^12^ Owolabi and Adejumo^12^ further explained that despite the high level of knowledge about cervical cancer screening and its advantages, nurses in some contexts have reportedly adopted and used cervical cancer screening services at relatively low rates. This poses an even more significant threat to the healthcare delivery system. Additionally, Oyekale et al.^13^ indicated that nurses comprise the majority of healthcare professionals and are essential resources for any country. Therefore, maintaining their health and safety is vital in providing ongoing patient care. Hence, this study aimed to explore primary health care (PHC) nurses’ lived experiences as they conduct screening for patients. It is deemed necessary, then, for a successful cervical cancer screening programme for the general population that the service providers’ lived experiences are explored. These experiences can potentially impact their subsequent screening or their goal of offering such services to their patients. Moreover, to our knowledge, there are no Lesotho-published studies regarding PHC nurses’ lived experiences of cervical cancer. Hence, this qualitative study addresses this knowledge gap. It is envisaged that the findings from this study may be necessary for developing interventions aimed at improving cervical cancer screening programmes among nurses. Moreover, improving cervical cancer screening among nurses could result in improved screening rates in the general population.

## Research methods and design

### Study design

This is a qualitative inquiry in which a phenomenological approach was used. According to Alase,^14^ phenomenology is a design used to understand the context of the lived experiences of people (research participants) and the meanings of their experiences. For this study, qualitative inquiry was most appropriate. According to Butler et al.,^15^ qualitative inquiry aims to discover new phenomena when the already existing theory that describes these phenomena is insufficient, or the theory is non-existent. Therefore, we conducted this type of study because, in Lesotho, cervical cancer screening has received a lot of attention among the general public, but little is known about screening among nurses, not to mention their lived experiences with screening.

### Study setting

The Leribe district, where this study was conducted, is the second most populated district in Lesotho, with a population of 298 352.^16^ There are 25 primary health care centres (PHCCs) in the district. All these facilities offer cervical cancer screening services. As per the Lesotho Guidelines for Screening and Treatment of Cervical Pre-Cancer, patients identified at these health centres as suspected cases of cervical cancer are referred to the hospital for management, including biopsy and staging.

### Participants’ profile

The participants in this study were female nurses employed in PHCCs in one of the districts in Lesotho. Their ages ranged between 29 years and 62 years. Four of them were nursing assistants; five were nursing sisters and one was a nursing officer. All had undergone cervical cancer screening as the study aimed to explore the participants’ lived experiences of cervical cancer screening.

### Participant selection

This study’s unit of analysis was the PHC nurses who had undergone cervical cancer screening; that is, male nurses and female nurses who had never undergone cervical cancer screening tests were excluded. To invite female nurses who were willing to participate in this study, the study’s information sheet, which included invitations to participate, was emailed to the District Health Management Team (DHMT) to distribute or send to all female nurses within the district. Then, the researcher called PHCCs in the nearby area to enquire about the availability of nurses interested in participating in the study and scheduled appointments for interviews with those nurses. On the scheduled interview days, the researcher interviewed eligible, available and willing nurses to participate. Two to three participants were selected for each of the four identified facilities. The researcher conducted interviews in nearby PHCCs and then moved to distal PHCCs. Thus, this study employed a purposive sampling technique, particularly the maximum variation sample, where the participants with various work experiences, training, skills and different age groups were selected.

### Data collection and analysis

In-depth, face-to-face, semi-structured interviews were conducted using an interview guide with a reliable audio recorder, enabling the researcher to observe non-verbal behaviour. The semi-structured interview guide enabled the researcher to pose spontaneous questions, follow-up questions and probe for more information. Interviews lasted between 20 min and 30 min, and participants granted permission to be recorded.

While the interviews were conducted in English, the participants were allowed to respond in Sesotho, which is their home language, and were afforded the expression of their choice. The researcher transcribed and translated the audio from Sesotho to English. The transcribed data were then analysed using thematic analysis, using the following steps as explained by Maguire and Delahunt.^17^ The steps include becoming familiar with the data, generating the initial codes, searching for themes, reviewing themes, defining themes and writing up. Thus, the process of data analysis in this study began with repeatedly listening to, translating and transcribing the recorded interview transcripts. Once the data were compiled and organised, the chunks of data were coded, where the initial codes were put into meaningful categories.

The coding process resulted in the discovery of potential themes. The themes were then reviewed and refined based on the relevant and supporting data from the transcripts. After the themes had been finalised, they were further divided into sub-themes, which provided more insight into the basic concepts of the main themes as well as the meaning these sub-themes expressed. Finally, the themes and sub-themes were thoroughly discussed among the two researchers until consensus was reached.

### Measure of trustworthiness

Ahmed^18^ explains that because of the subjective nature of qualitative findings, it is vital to ensure trustworthiness in establishing the reliability and credibility of qualitative findings. Haq et al.^19^ further explain that credibility, transferability, dependability and confirmability of research design, process and action can all be used to measure how trustworthy a qualitative study is.

Credibility is described as the extent to which the findings correctly reflect the truth about what the participants went through.^18^ To ensure credibility, member checking was used. Member checking was done by going back to some of the participants after the data were analysed and interpreted, so the participants could confirm whether they were correctly interpreted. To maintain the study’s credibility, we also utilised exact excerpts from the transcripts while presenting the findings.

Transferability refers to the extent to which qualitative research findings can be transferred to other settings with different respondents.^18^ Transferability was ensured through the provision of a thick description, that is, sufficient details about the setting, sample characteristics, data collection and analysis methods. The study’s use of purposive sampling, which would also allow other researchers to concentrate on reliable sources who are especially knowledgeable about the topics under investigation, further guaranteed transferability.

Dependability refers to the consistency of results over comparable contexts.^19^ According to Haq et al.,^19^ if a study’s findings can be repeated in a setting or population with similar characteristics, then the study’s findings are considered dependable. Furthermore, to ensure a study’s dependability, Haq et al.^19^ assert that having an expert opinion is highly beneficial in enhancing data collection methods, processes, analysis plans and study interpretation. Therefore, from the beginning until the end of this project, the principal investigator, who has training in qualitative research, was guided by an experienced supervisor to ensure dependability.

Confirmability aims to ensure that the study’s findings are not impacted by any biases or preferences of the researcher.^18^ Confirmability in this study was ensured using a reflexive journal in which the researcher’s preconceived ideas about the research topic were noted, so that they did not interfere with the interpretations of the findings.

### Ethical considerations

Ethical clearance to conduct this study was obtained from the University of KwaZulu-Natal Biomedical Research Ethics Committee (BREC/00006211/2023).

## Results

The findings are presented in relation to an adapted version of the socio-ecological model (SEM) in Table 1. They are mapped against five constructs of SEM as defined by Lang et al.,^21^ namely: (1) intrapersonal factors, which refer to an individual’s characteristics such as knowledge, attitudes, self-concept, and so on; (2) interpersonal factors, which refer to social networks and social support systems such as family, work colleagues and friends; (3) institutional factors (health system), which are those that relate to organisational characteristics and (4) community factors, which relate to relationships among organisations and institutional and informal networks. The SEM is used in this study to emphasise the interaction of different factors (in this case, the experiences) with nurses’ decisions to undergo screening routinely.

**TABLE 1:** Summary of themes and sub-themes derived from the analysis of the primary health care nurses’ lived experiences of cervical cancer screening.

Main themes	Sub-themes
1. Intrapersonal experiences	1.1. Feelings of embarrassment 1.2. Capacity to handle positive results 1.3. Intrusion of privacy vs procedure acceptability
2. Interpersonal experiences	2.1. Health worker–health worker relationship 2.2. Freedom of choice
3. Institution-related experiences	3.1. Good nursing services vs poor counselling services 3.2. Prolonged turnaround time of results 3.3. Unconducive work environment 3.4. Prevention of pain vs discomfort during the procedure
4. Community-related experiences	4.1. Increased number of cervical cancer deaths observed

*Source:* Johnson M, Wakefield C, Garthe K. Qualitative socioecological factors of cervical cancer screening use among transgender men. Prev. Med. Rep. 2020;17:101052. https://doi.org/10.1016/j.pmedr.2020.101052^20^

Table 2 presents the demographic composition of the study sample. The ages of the participants ranged from 29 years to 62 years, and the years of work experience ranged from 4 years to 38 years. Similarly, the participants’ designations varied.

**TABLE 2:** Demographic characteristics of the participating primary health care nurses.

Participant	Nursing cadres	Age in years	Years of experience
001	Nursing assistant	62	38
002	Nursing sister	43	8
003	Nursing sister	30	4
004	Nursing sister	29	7
005	Nursing assistant	53	15
006	Nursing assistant	45	13
007	Nursing assistant	39	15
008	Nursing officer	50	19
009	Nursing sister	53	31
010	Nursing sister	48	23

### Theme 1: Intrapersonal experiences

The participants were asked about their experiences regarding cervical cancer screening, where they would describe their experiences with screening and how they felt about them. The following categories represent the sub-themes that emerged from the intrapersonal experiences.

#### Sub-theme 1.1: Feelings of embarrassment

Most participants reported feeling embarrassed during the procedure:

‘As a person, I was embarrassed to open [*the legs*] so the procedure could be done. Even though I am a nurse and know how this procedure is done, especially because I was screened by a man, being like that was embarrassing.’ (Participant 009, 53 years, nursing sister)

As can be seen from the statement above, some participants find it embarrassing to have their examinations performed by a male HCW. Conversely, other participants noted that it is embarrassing to be screened by their colleagues. Thus, being screened by their colleagues is the issue, not whether a nurse is male or female. Participant 004 emphasised this by saying:

‘I have realised that even these Basotho women only go for screening because we beg them. It is not easy to get naked in front of another person, and it is even worse if the other person is your colleague. I don’t want to get naked in front of my colleagues.’ (Participant 004, 29 years, nursing sister)

Participants also cared about the screeners’ age:

‘… the negative experience was that the person conducting my screening was young.’ (Participant 008, 50 years, nursing officer)

Therefore, for future screening, participants expressed a preference for screeners who are older:

‘I will not be screened by children …’ (Participant 007, 39 years, nursing assistant)

‘Yes, but only if I am screened by an older person.’ (Participant 009, 53 years, nursing sister)

#### Sub-theme 1.2: Capacity to handle positive results

Participants were also anxious about what the results would show. To underscore this, here is what two participants said:

‘I felt I would not survive receiving positive results.’ (Participant 001, 62 years, nursing assistant)

‘I wondered what would happen should I be told that I have cancer. The thought of positive results makes me anxious.’ (Participant 002, 43 years, nursing sister)

#### Sub-theme 1.3: Intrusion of privacy vs procedure acceptability

Most participants did have their subsequent screening tests, but they did so long after the due dates. Past screening experiences, such as a lack of privacy, may be the cause of this, as articulated by participants:

‘Yes, indeed, I will go for screening after some time; I won’t go at the expected time … Seriously, no! As for the other person looking at my private parts, it is a no. Even if it is someone you know or someone you don’t know, the fact remains that they are going to look at your private parts. So, no!’ (Participant 008, 39 years, nursing assistant)

‘There is no privacy there because while I was in lithotomy position, other people kept coming in.’ (Participant 010, 48 years, nursing sister)

There might be fewer screening recommendations and less education for the public by these health professionals who understand the importance of screening yet prefer to postpone the planned screening because of these concerns. However, not every participant had concerns about their private parts being exposed, as articulated by one participant:

‘It was a positive experience because I was not bothered by the procedure. I can do it again and again, I don’t mind.’ (Participant 001, 62 years, nursing assistant)

### Theme 2: Interpersonal experiences

#### Sub-theme 2.1: Health worker-health worker relationship

Even though some participants expressed feelings of embarrassment when screened by their colleagues, for some, being screened by coworkers was a motivating element to get screened:

‘… and one of the days in the morning, I prepared the equipment and called my colleague to come and see. When she arrived, I asked her to screen me for cervical cancer. She was surprised but was obliged to act because the table was fully equipped.’ (Participant 006, 45 years, nursing assistant)

‘Yes. So, the person who was examining me, I had started with her first … I said no, I can’t miss the opportunity of being screened, yet there are only the two of us in here. I am climbing that bed immediately when you get down.’ (Participant 005, 53 years, nursing assistant)

#### Sub-theme 2.2: Freedom of choice

Some of the participants expressed preferences in terms of screening methods. Thus, they were granted the opportunity to choose what suited them the most:

‘What I wanted was to get screened with disposable speculums, not reusable ones, to prevent infection. I asked her to use VILI for me, not VIA. She used VILI because I realised that it is reliable.’ (Participant 004, 29 years, nursing sister)

Other participants noted how they were at liberty to exercise their choice of a healthcare provider:

‘I had already asked for a nurse that I thought I would be comfortable with.’ (Participant 010, 48 years, nursing sister)

In addition to choice, the participants were also at liberty to confirm their findings:

‘I wanted her to just give me the results and take a picture so I could see myself. I asked her to use iodine so I could see that my cervix was black and not mustered yellow. Also, if she used VIA, I wouldn’t be able to interpret my results from the photo I asked her to take.’ (Participant 004, 29 years, nursing sister)

This suggests that if nurses do not take responsibility for deciding who should conduct their screenings and how to carry them out, or if they do not take a self-directed approach, there is a strong likelihood that the screenings will not be done. Two participants emphasised this point further:

‘I am never taking the test again until the HPV test is available, then I can conduct my screening …’ (Participant 004, 29 years, nursing sister)

‘I felt that screening is important, even though I did not go back because my nurse was no longer there …’ (Participant 010, 48 years, nursing sister)

In summary, these interpersonal experiences indicate that, unlike their patients, nurses may have certain advantages when it comes to cervical cancer screening because they are at liberty to assert their rights as patients.

### Theme 3: Institution-related experiences

#### Sub-theme 3.1: Good nursing services vs poor counselling services

During their screening process, the participants cited that they were provided with good nursing services:

‘I was treated well, I was given health education, and they ensured that the room was conducive and confidential … the equipment was in good condition, the speculum was warm enough to prevent irritation.’ (Participant 002, 43 years, nursing sister)

Another participant reflected on the post-screening process and noted:

‘When the results came back, they sat me down so I wouldn’t worry too much about them (results) and could accept whatever they showed.’ (Participant 001, 62 years, nursing assistant)

Although some participants reported receiving health education or an explanation of the procedure before being screened, one participant stated that she never received any explanation:

‘When health professionals conduct our screening or for other health professionals, they say we already know, so they don’t even give us counselling.’ (Participant 004, 29 years, nursing sister)

#### Sub-theme 3.2: Prolonged turnaround time of results

Among the techniques mentioned by the participants, the pap smear was thought to be the most effective:

‘… they did a pap smear whose results never came back. I think the only thing negative was not getting the results, which leaves you feeling like you did nothing to know if you have the disease or not.’ (Participant 009, 53 years, nursing sister)

Not only did the long turnaround time of the results or not receiving the results make the participants feel like they neglected their health, but they were also anxious about what the results would eventually show. To underscore this, participant 006 said:

‘… after taking the test, I was stressed about my results and wondered how I would feel if they came back positive. That’s why I wanted them out urgently to escape the stress of waiting …’ (Participant 006, 45 years, nursing assistant)

#### Sub-theme 3.3: Unconducive work environment

One participant reported inadequacy in the efficacy of the interventions to address the sterility of the air between the procedures:

‘The other one is the smell in the room, which is offensive, and the spraying of air freshener in between the patients made me question myself if the bad smell came from me or the person who was screened before me.’ (Participant 008, 39 years, nursing sister)

#### Sub-theme 3.4: Prevention of pain vs discomfort during the procedure

Most participants described cervical cancer screening as painful based on the observations they made as they screened other women or observed the procedure done to other women. But for them, they reported that all that could be done to prevent pain was done:

‘I did not feel anything that everyone has been saying about the pain. I had no problem with the speculum because it was warm and lubricated …’ (Participant 002, 43 years, nursing sister)

‘… but this time around, there was no discomfort. I think it’s because the procedure was explained, and the screeners were instructed to select speculum sizes based on the patient’s body size. Also, they were told not to tilt the speculum but to go just straight and open it.’ (Participant 003, 30 years, nursing sister)

While other participants described strategies to prevent pain when screening for cervical cancer, some participants mentioned pain as one of the discomforting feelings they experienced:

‘I just felt that inserting a speculum in there was painful, especially when it was opened. Hey, it is very painful, even if it is a plastic speculum, it is not comfortable at all … when Lugol’s iodine encounters the vaginal walls, you will feel like you are burning … I experienced burning pain; I can’t even explain that pain.’ (Participant 004, 29 years, nursing sister)

### Theme 4: Community-related experiences

#### Sub-theme 4.1: Increased number of cervical cancer deaths observed

In addition to their personal lived experiences, participants also shared experiences from caring for their communities:

‘I feared cancer and noticed that people who have cancer suffer a lot, so I did not want to suffer.’ (Participant 001, 62 years, nursing assistant)

‘Maybe it is because we have seen so many women with cancer at certain stages, some having to go under hysterectomy. So, I don’t want to find myself in such situations.’ (Participant 003, 30 years, nursing sister)

## Discussion

This section discusses the study’s findings in relation to the literature and the research objective. The study focused on exploring PHC nurses’ lived experiences of cervical cancer screening in the Leribe district, Lesotho. Four dominating themes emerged from the data: intrapersonal experiences, interpersonal experiences, institution-related experiences and community-related experiences. Below is a brief analysis of participants’ ages, work experiences and educational backgrounds in relation to cervical cancer screening.

The participants who were nursing sisters aged 30 years and under and had less than 10 years of experience seemed to have more knowledge of cervical cancer screening. However, even with that knowledge, they only had one screening instead of at least two, as the guidelines state that screening starts at age 25 years and is then repeated every 2–3 years, depending on the patient’s human immunodeficiency virus (HIV) status.^22^ As this study’s sample size was small, a bigger study with a larger sample size may be needed to test whether the nurses’ knowledge of cervical cancer has a bearing on their desire to get screened routinely. Also, the older participants, those aged over 30 years with more than 10 years of work experience, had more than one screening test done compared with the younger participants with less than 10 years of work experience. These findings are almost comparable to those of a study done in Nigeria with participants under 30 years of age and who had practised for 5 years or less.^23^ That study concluded that because the nurses were young, they did not seem to think they would develop precancerous or cancerous lesions and were therefore not concerned about such matters, which made them less likely to use the service. Moreover, a study conducted in Turkey^24^ found that among nurses and midwives, the frequency of pap smear tests increased greatly with age. Thus, the present study found that nurses’ decisions to get screened for cervical cancer were influenced by their age and years of experience.

The findings of the study were mostly consistent with most previous research among nurses. However, these studies did not report on cervical cancer screening among nurses, but rather on when nurses needed medical attention. For example, the study by Shattnawi et al.^25^ indicated that nurses in that study reported receiving special treatment from other healthcare providers simply because they were nurses. Other studies indicated that nurses seeking care recognised the profound impact of seemingly minor interactions and gestures that made them feel genuinely supported, respected and cared for during their medical treatment.^26,27^ Furthermore, there is a shortage of studies on nurses’ participation in cervical cancer screening. Most research demonstrates the results of women in the general population. Consequently, a lack of literature addressing this topic means that little is known about nurses’ experiences with cervical cancer screening. However, in this study, nurses reported their lived experiences of cervical cancer screening. Bigger studies may be needed to further explore these experiences. The exceptional care that included health education and well-maintained equipment to prevent pain was one of the experiences that the nurses highlighted. These limited experiences indicate that PHC nurses follow the cervical pre-cancer and treatment guidelines, which state that the speculum should be lubricated and warmed prior to the procedure and that the patient must be informed about the procedure.^22^ The participants also appreciated the best nursing services that they received during the screening process. They valued that they were treated as patients, not experts. Even though they desired and valued the patient treatment they received, their expertise worked in their favour as they were able to exercise their patient rights of choosing their healthcare provider, the screening technique and the equipment that suited them best. When these nurses are content with their choices of screening techniques, equipment and screeners, they may also have honest conversations with their patients to empower them to know and exercise their rights. This was emphasised by Hunt and Buckley^27^ in their study, which revealed that the personal experiences the nurse-patients had with their carers (nurses) made them explore and address potential problems in clinical practice to provide better quality care for patients. The experiences reported in this study aligned with the findings of a study conducted by de Queiroz Neves et al.,^28^ which revealed that participants found the screening to be comfortable because of the nurses’ provision of a relaxing atmosphere and their extreme kindness and respect.

According to the Lesotho cervical cancer screening guidelines, the screening procedure involves a brief general examination that entails assessing for pallor, blood pressure, breast examination and speculum examination. A speculum is explained by Taylor et al.^29^ as a medical instrument that has two blades with a hinged joint that allows it to be opened and fixed into position, allowing access and visualisation of the vagina canal, cervix and uterus. The participants in this study explained that screening with a speculum was an invasion of their privacy because their private parts were exposed, not only to their screeners but also to everyone who kept coming in while they were in that position. These findings are almost the same as the findings of Sarcheshme and Mahdizadeh’s^21^ study, which reported that the healthcare providers felt embarrassed and ashamed of the internal examination, which revealed their intimate organs, and the position required for sampling. Due to these concerns, the participants in this study highlighted and expressed a preference for another technique, the Human Papilloma Virus (HPV) test, which requires self-sampling. The participants in a Ghanaian study, who were female HCWs who took the sample themselves, expressed similar feelings.^30^ They showed a preference for the HPV test as they thought it was comfortable, and 82.7% of them said they would get screened more frequently with this test. Furthermore, they would suggest screening with this test to their patients, relatives and neighbours. Behnke et al.^30^ further explained that less pain compared with a speculum examination and more privacy were the most cited reasons for preferring self-sampling.

In contrast to the other participants who reported receiving excellent care, such as an explanation of the process, some participants reported negative experiences, such as not receiving counselling prior to the process. Their screeners assumed that because the participants were nurses, they had knowledge of cervical cancer screening and, therefore, needed no counselling. These findings are congruent with those of a study conducted by Hunt and Buckley,^27^ which revealed that nurses often do not give enough information to their nurse-patients regarding their treatment because they assume that nurse-patients are familiar with their illnesses. The participants in this study also expressed stress over the possible outcomes of the results, especially with pap smears, as they took too long to return or never received the results at all. Failure to get the pap test results could indicate the inadequacy of the health system, which could discourage other women, especially those in the general public, from screening routinely, as they might not be aware of these alternatives. This was reinforced by the Bolivian study, which showed that women were disappointed over not receiving their pap test results and so disregarded the need to continue screening.^31^ Thus, if cervical cancer screening uptake or coverage is to improve, then this is a major area of improvement within the health system.

Feelings of uneasiness, such as pain, embarrassment, an unpleasant screening environment and being screened by their colleagues, were among the experiences cited by the participants. These findings are similar to the findings of a study conducted by Suantika et al.,^10^ which indicated that nurses in that study experienced pain and discomfort on examination and had feelings of shame, especially with the opposite-sex examiner. Contrary to the previous research, the majority of this study’s participants were more concerned about being screened by their coworkers or junior colleagues than they were about being screened by the opposite-sex examiner. This could be due to the best performance of senior nurses. Lewaherilla et al.^32^ agree with this, as they explained that senior nurses are more knowledgeable and wiser because of the experience they gained over the years. Lewaherilla et al.^32^ explained seniority in terms of age, years of experience and length of service. Thus, further research on nurses’ preferences for a screener might be necessary. Based on McDowell et al.’s^33^ findings of a predominantly female nursing workforce, potential screening protocols could recommend sex-matched examinations, contingent upon the research findings supporting existing literature that suggests healthcare professionals may experience discomfort when screened by examiners of the opposite sex. The present study’s findings also concur with the results of a study conducted by Sarcheshme et al.,^34^ which showed that negative past experiences act as deterrents to taking a pap smear, affecting people’s willingness to repeat the screening. The deterrents mentioned by the participants in that study included inappropriate physical settings, low quality of sampling, distrust in results and lengthy delays in providing the test results. Despite the negative experiences reported in this study, some participants were confident enough to take the subsequent test or were willing to retest in the future and suggest screening to other women. The results of this study, therefore, demonstrate that while some nurses’ intentions to screen again in the future appeared to be adversely impacted by their negative experiences, their service delivery objective of encouraging other women to get screened for cervical cancer remained unaffected.

### Limitations

As this study was self-funded and conducted by a full-time employed student, the interviews were only conducted among nurses working in PHCCs located in lowland areas. Therefore, the findings of this study may be transferable to similar urban settings. Hence, larger studies with PHC nurses in the country could be conducted in the future to explore this topic in-depth for greater applicability.

### Recommendations

Based on the study findings, the authors recommend the following. Firstly, since most nurses were inspired to detect cervical cancer from what they learned about it and how they could reduce pain during the procedure, regular cervical cancer training is recommended among HCWs. Also, not only did those trainings inspire the nurses to get screened, but they also inspired them to offer screening services to all women, including those who come for family planning services only. Thus, we recommend that this practice of offering screening services to all women, regardless of their reasons for visiting health facilities, be adopted as a standard practice in the health system. Secondly, interventions to improve the turnaround time of the results should be explored, as one of the factors that deterred nurses from screening was the waiting period for the results. Thirdly, less intrusive screening modalities should be investigated to alleviate the discomfort caused by existing methods of cervical cancer screening. One of these less intrusive screening modalities, HPV testing, was only introduced in the Leribe district months after data collection. Therefore, we recommend further studies on how to improve the screening experience, as the new Guide for HPV test^35^ explains that with a positive HPV test, triage tests for treatment using visual inspection with acetic acid (VIA), visual inspection using Lugol’s iodine (VILI) and pap smear are recommended.

## Conclusion

The study findings provided important insights into PHC nurses’ lived experiences of cervical cancer screening. This evidence is important for the amendment and redesigning of cervical cancer screening programmes to improve cervical cancer screening uptake among nurses, who may have more honest conversations with other women based on their positive experiences and hence be able to recommend screening to other women. Furthermore, even with the self-sampling HPV test, the recently implemented guidance for HPV tests and management for the prevention of cervical cancer in Lesotho^35^ recommends triage tests following positive HPV tests. These include VIA, pap smear and others. Therefore, improving the cervical cancer screening experience with VIA, VILI and pap smear is a promising approach to improve cervical cancer screening uptake. Thus, further studies on how to improve the screening experience are recommended.
